# Supplementary material for: Loss of primary cilia and dopaminergic neuroprotection in pathogenic LRRK2-driven and idiopathic Parkinson’s disease
Source: bioRxiv. 2024 Jan 16:2024.01.15.575737. Preprint. [Version 1] doi: 10.1101/2024.01.15.575737 (PMC10827083; doi:10.1101/2024.01.15.575737)
Supplement: Supplement 2 [file NIHPP2024.01.15.575737v1-supplement-2.pdf]

bioRxiv preprint doi: https://doi.org/10.1101/2024.01.15.575737; this version posted January 16, 2024. The copyright holder for this preprint (which was not certified by peer review) is the author/funder, who has granted bioRxiv a license to display the preprint in perpetuity. It is made available under aCC-BY 4.0 International license.

Supplemental Table 1. This Excel file reports the differentially expressed genes from comparisons of G2019S LRRK2 knockin (KI) and wildtype nuclei from the mouse dorsal striatum in our single nucleus RNA sequencing dataset. This file is also available on Dryad (https://doi.org/10.5061/dryad.pk0p2ngvp). Individual sheets in the file report the results for a given cell type cluster/subcluster as indicated. Differentially expressed genes were identified using the Seurat "FindMarker()" command in RStudio. The columns list gene names (**Names**), p values (**p_val**), log2 fold changes (**log2FoldChange**), percentage of nuclei from G2019S KI mice expressing the listed gene (**pct.1**), percentage of nuclei from WT mice expressing the listed gene (**pct.2**), adjusted p values (**padj**), number of reads obtained from G2019S KI mice for the listed gene (**Counts_GS**), and the number of reads obtained from WT mice for the listed gene (**Counts_WT**).

Supplemental Table 2. Key Resources used in this study.

Supplemental Table 3 Top 10 gene list for each astrocyte and cholinergic interneuron subcluster.

bioRxiv preprint doi: https://doi.org/10.1101/2024.01.15.575737; this version posted January 16, 2024. The copyright holder for this preprint (which was not certified by peer review) is the author/funder, who has granted bioRxiv a license to display the preprint in perpetuity. It is made available under aCC-BY 4.0 International license.

Table S2. **Key Resources used in this study**

Reagent type (species) or resource	Designation	Source or reference	Identifiers	Additional information
Genetic reagent(Mus musculus)	Constitutive KI Lrrk2tm4.1Arte	Taconic	#13940, RRID:IMSR_TAC:13940	C57BL/6; G2019S KI
Genetic reagent(Mus musculus)	Ppm1h-/- mouse	Taconic	#TF3142	C57BL/6 Background
Antibody	anti-Choline Acetyltransferase (goat polyclonal)	Millipore	AB144P-1ML (RRID:AB_2079751)	(1:200)
Antibody	anti-Adenylate cyclase III (mouse monoclonal)	Santa Cruz	SC-518057 (RRID:AB_3073967)	(1:100)
Antibody	anti-DARPP-32 (rabbit monoclonal)	Cell Signaling Technology	#2306S (RRID:AB_823479)	(1:400)
Antibody	anti-GFAP (chicken polyclonal)	EnCOR	CPCA-GFAP (RRID:AB_2109953)	(1:2000)
Antibody	anti-Arl13B (mouse monoclonal)	Neuromab	N295B/66 (RRID:AB_2877361)	(1:500)
Antibody	anti-GFR alpha-1/GDNF R alpha-1 (goat polyclonal)	R&D Systems	AF560 (RRID:AB_2110307)	(1:500)
Antibody	anti-Tyrosine hydroxylase (sheep polyclonal)	Novus Biologicals	NB300-110 (RRID:AB_10002491)	(1:500)
Antibody	anti-Cntn5 (rabbit polyclonal)	Novus Biologicals	NBP1-83242 (RRID:AB_11019867)	(1:50)
Antibody	anti-NeuN(chicken polyclonal)	Millipore	ABN91 (RRID:AB_11205760)	(1:1000)
Antibody	anti-DRD2(mouse monoclonal)	NeuroMab	N186/29 (RRID:AB_11000721)	(1:250)
Antibody	H+L Donkey anti-mouse Alexa 488	Life Technologies	A32766 (RRID:AB_2762823)	(1:2000)
Antibody	H+L Donkey anti-Rabbit Alexa 568	Life Technologies	A10042 (RRID:AB_2534017)	(1:2000)
Antibody	H+L Donkey anti-goat Alexa 488	Life Technologies	A11055 (RRID:AB_2534102)	(1:2000)
Antibody	H+L Donkey anti-mouse Alexa 647	Life Technologies	A31571 (RRID:AB_162542)	(1:2000)
Antibody	H+L Donkey anti-chicken Alexa 488	Jackson ImmunoResearch	#703-545-155 (RRID:AB_2340375)	(1:2000)
Antibody	H+L Donkey anti-sheep Alexa 488	Life Technologies	A-11015 (RRID:AB_2534082)	(1:2000)
Antibody	H+L Donkey anti-goat Alexa 568	Life Technologies	A-11057 (RRID:AB_2534104)	(1:2000)
Reagent	Sudan Black B	Chem-Impex International	#01307	
Commercial assay or kit	RNAscopeMultiplexFluorescentReagent Kit v2	Advanced Cell Diagnostics	#323100	
Commercial assay or kit	RNAscope Probe- Mm-Lrrk2	Advanced Cell Diagnostics	#421551	(1:20)

bioRxiv preprint doi: https://doi.org/10.1101/2024.01.15.575737; this version posted January 16, 2024. The copyright holder for this preprint (which was not certified by peer review) is the author/funder, who has granted bioRxiv a license to display the preprint in perpetuity. It is made available under aCC-BY 4.0 International license.

Commercial assay or kit	OPAL 690 REAGENT PACK	Akoya Biosciences	FP1497001KT	
Software, Algorithm	FIJI	PMID:29187165	RRID:SCR_002285	
Software, Algorithm	CellProfiler	PMID:29969450	RRID:SCR_007358	
Software, Algorithm	Prism	Prism 9.3.1 (350)	RRID:SCR_002798	
Software, Algorithm	RStudio	https://posit.co/	RRID:SCR_000432	
Software, Algorithm	Seurat	PMID:29608179	RRID:SCR_016341	
Software, Algorithm	ImageJ	https://imagej.net/	ImageJ RRID:SCR_003070	
Software, Algorithm	DoubletFinder	PMID:30954475	RRID:SCR_018771	

Supplemental Table 3.

Table 3A. Top 10 gene list for each astrocyte subcluster.

Cluster ID	Gene List
Aldh1a1	Aldh1a1, Fry, C730002L08Rik, Unc13c, Cntn1, Luzp2, Trpm3, Egfem1, Gpr158, Otud7a
Crym	Crym, Pde10a, Robo1, Zswim6, Gabbr2, Pde7b, Hdac4, Gm12239, Ncam2, Hes5
Nrg1	Nrg1, Adamts18, Csmd1, Slit2, Ldb2, Nkain2, Meg3, Adgrv1, Tox3, Prr16
Gfap	Igfbp5, Thbs4, Kcnj3, Prkca, Clu, Garnl3, Gfap, Apoe, Gja1, Aqp4

Table 3B. Top 10 gene list for each cholinergic interneuron subcluster.

Cluster ID	Gene List
Elavl2	Elavl2, Luzp2, Inpp4b, Tacr1, Asic2, Slc7a14, Clstn2, Cpne4, Trpc3, Rgs6
Grm5	A330015K06Rik, Grm5, Ptprd, Kcnq5, Pde10a, Pde7b, Rarb, Meis2, Cntn5, Plcb1
